# A nationwide analysis of mortality trends due to hypertensive heart disease with co-existing behavioral and mental disorders (1999–2020)

**DOI:** 10.1097/MS9.0000000000004746

**Published:** 2026-01-27

**Authors:** Warda Imran, Sarim Hassan Shahab, Hafsa Rafique, Siddique Ahmad, Saad Ahmad Idrees, Maria Baig, Raveen Muzaffer, Abubakar Nazir, Muhammad Ahmad, Muhammad Kashan, Imran Naqvi, Ghulam Mujtaba Ghumman, Ahmed Jamal Chaudhary, Muhammad Saad, Naseer Khan, Syed Sohail Ali

**Affiliations:** a Department of Medicine, Dow Medical College, Karachi, Pakistan; b Department of Internal Medicine, Nishtar Medical University, Multan, Pakistan; c Department of Internal Medicine, FMH College of Medicine and Dentistry, Lahore, Pakistan; d Department of Medicine, Nishtar Medical University Multan, Multan, Pakistan; e Department of Medicine, Khyber Medical College, Peshawar, Pakistan; f Department of Physiology, Dow Medical College, Karachi, Pakistan; g Department of Internal Medicine, Dow Medical College, Karachi, Pakistan; h Department of Internal Medicine, The Jewish Hospital – Mercy Health, Cincinnati, OH, USA; i Department of Medicine, Oli Health Magazine Organization, Kigali, Rwanda; j Department of Physiology, Nishtar Medical University, Multan, Pakistan; k Department of Internal Medicine, Mercy Health Saint Vincent Medical Center, Toledo, OH, USA; l Department of Medicine, DMC Sinai-Grace Hospital, Michigan, Detroit, MI, USA; m Department of Medicine, University of Cincinnati, Cincinnati, OH, USA

**Keywords:** age-adjusted mortality rate, behavioral and mental disorders, hypertensive heart disease, mortality trends, racial disparities, regional disparities

## Abstract

**Introduction:**

One of the leading causes of cardiovascular death is hypertensive heart disease (HHD), especially in people who also have behavioral and mental disorders (BMDs). Cardiovascular risk is increased in this population by lifestyle factors, drug side effects, and inflammatory pathways. National data on HHD mortality trends for impacted subgroups in the USA are still scarce, despite the growing burden of both BMDs and HHD.

**Methods:**

Using the CDC WONDER Multiple Cause of Death database, we examined death records from 1999 to 2020 in the USA. ICD-10 codes for BMDs (F01–F99) and hypertensive cardiac disease (I11.0–I11.9) were used to identify cases. Regional and demographic-specific age-adjusted mortality rates (AAMRs) were computed. Annual percent changes (APCs) and trends were assessed using joinpoint regression, with significance set at *P* < 0.05.

**Results:**

About 284 797 people died from HHD with accompanying mental illnesses between 1999 and 2020. From 1.15 to 13.69 per 100 000, the AAMR increased (AAPC: 11.65%; *P* < 0.0001). AAMRs peaked among Black adults and were consistently higher in men. Regional load was largest in the South, and final fatality rates were higher in urban than rural areas. Disparities at the state level varied from 1.40 in Nebraska to 15.71 in Washington, DC. The greatest increases in HHD-related mortality were linked to male sex, substance-related mental illnesses, and living in underserved areas.

**Conclusion:**

Men, Black adults, and Southern regions were disproportionately affected by the more than 10-fold increase in mortality from HHD and mental disorders between 1999 and 2020. Urban areas saw the largest increases, underscoring the urgent regional and demographic disparities that require focused interventions.

## Introduction

Hypertensive heart disease (HHD) is a medical condition marked by various pathological changes in the heart, mainly impacting the left ventricle. It arises from sustained high blood pressure and is linked to several symptoms, including myocardial hypertrophy, arteriosclerosis, and cardiac arrhythmias^[1]^. As the fourth most common cause of cardiovascular (CV) and cerebrovascular fatalities, HHD ranks just below ischemic heart disease, stroke, and cardiomyopathy^[2]^. Based on the 2019 Global Burden of Disease Study (GBD), there were 18.60 million instances and 21.51 million disability-adjusted life years (DALYs) attributed to HHD globally. HHD creates a significant health and economic strain, making it an important public health issue^[3]^. Although age-standardized DALY rates have decreased, the overall count of HHD cases has increased over the last 30 years^[4]^. Around 120 million adults in the USA (48.1%) are affected by hypertension; among them, 92.9 million (77.4%) struggle with uncontrolled hypertension^[5]^.

Behavioral and mental disorders (BMDs), which encompass major depressive disorder, anxiety disorders, bipolar disorder, schizophrenia, Attention deficit hyperactivity disorder (ADHD), and conduct disorders, represent a significant and increasing proportion of the global disease burden. The GBD 2019 reports that the total DALYs associated with BMD rose from 80.8 million in 1990 to 125.3 million in 2019, increasing from 3.1% to 4.9% of global DALYs, while the age-standardized DALY rates have remained consistent^[6,7]^.

People suffering from depression, bipolar disorder, and schizophrenia face a markedly increased risk of experiencing cardiovascular diseases (CVDs), such as HHD, due to influences like lifestyle choices and the side effects of medications^[8,9]^. This reciprocal relationship deteriorates clinical outcomes and leads to higher rates of CV mortality among those with psychiatric conditions^[10]^. Depression is linked to increased levels of inflammatory markers such as C-reactive protein (CRP), Interleukin-6 (IL-6), and Tumor Necrosis Factor-alpha (TNF-α), which play a role in endothelial dysfunction, arterial stiffness, and the rapid progression of atherosclerosis leading to HHD^[11]^. Second-generation antipsychotic medications (such as olanzapine and clozapine) lead to considerable weight gain, insulin resistance, and abnormal lipid levels, which elevate the risks of CV issues and hypertension^[12]^. Patients with psychiatric abnormalities tend to smoke three times more than the average^[13]^. A recent study indicated that there was a significant rise in both medical and external mortality rates from 1999 to 2019 in the USA among individuals dealing with substance abuse, with CV diseases identified as the primary medical cause of death in this group^[14]^. A substantial number of patients with substance use disorders also suffer from associated psychiatric disorders, which heightens their risk for CV issues^[15]^.

Sociodemographic variables exacerbate the differences in CV health experienced by people with psychiatric conditions, leading to earlier CVD onset in those with schizophrenia and depression, a higher risk in men, and significantly increased hypertension-related mortality among Black individuals and those living in underrepresented areas of the USA^[16,17]^. While prior research has largely focused on HHD or psychiatric conditions independently, and some studies have explored CV mortality in the context of comorbid psychiatric illness, there remains a lack of comprehensive analysis that specifically quantifies mortality trends in individuals with HHD complicated by coexisting BMDs over time. To our knowledge, this is the first nationwide, longitudinal analysis of mortality trends in patients with HHD coexisting with BMDs.

Understanding the trends and burden of HHD in patients suffering from BMDs across gender, ethnicity, and geographical locations will guide healthcare providers to strategize according to the need. Therefore, our article aims to analyze and describe national trends and disparities in mortality due to HHD and coexisting BMDs among patients aged 25 and older across various demographic and geographical locations in the USA.

## Methods

### Study design and data source

Mortality data were extracted for analysis from the Centers for Disease Control and Prevention wide ranging online data for epidemiologic research (CDC WONDER) database^[18]^, focusing on death records from 1999 to 2020. The information was retrieved from U.S. death certificates through the Multiple Cause-of-Death (MCOD) database, which incorporates records from all 50 states and the District of Columbia. We identified pertinent literature through purposeful PubMed and Google Scholar searches using various combinations of the terms “hypertensive disorders,” “mental and behavioral disorders,” and “United States.” Priority was given to peer-reviewed articles published in the last 20 years and national surveillance data to clarify the context for the background and gaps in the literature.

**HIGHLIGHTS**

- **Sharp mortality increase**: Between 1999 and 2020, age-adjusted mortality rates from hypertensive heart disease combined with mental disorders in the USA rose more than 10-fold from 1.15 to 13.69 per 100 000.
- **Persistent disparities**: Mortality rates were consistently higher in men, were highest among Black/African American adults, and showed pronounced regional inequalities, particularly in the Southern census region.
- **Urgent public health need**: Both urban and rural areas experienced alarming increases, underscoring the need for integrated, targeted interventions and enhanced screening to address this growing burden.

### Study population

Mortality records were identified using International Classification of Diseases, 10th Revision (ICD-10) codes specific to HHD (I11.0-I11.9)^[19]^. BMDs were identified using codes (F01–F99)^[20]^. Only those records were included in the study that listed both the HHD and BMD codes among multiple causes of death.

#### Data abstraction

The study variables extracted for analysis encompassed year of death; demographic attributes including age, sex, and race/ethnicity; location and setting of death; geographic region; 10-year age groups; and urban–rural classification. Place of death was grouped into medical facilities (including outpatient, emergency department, inpatient, dead on arrival, or unknown status), home, hospice, and long-term care facilities. Race and ethnicity were categorized as non-Hispanic (NH) White, NH Black, and Hispanic; NH American Indian or Alaskan Native and NH Asian or Pacific Islander groups were grouped as NH Others. Ten-year age group variable was used to include adults aged ≥25 years and older. Urban-rural status was determined using the National Center for Health Statistics Urban-Rural Classification Scheme based on 2013 U.S. Census data^[21]^. Geographic regions were defined in accordance with the U.S. Census Bureau’s regional designations (Northeast, Midwest, South, and West).

### Statistical analysis

Age-adjusted mortality rates (AAMRs) per 100 000 population were estimated by year, sex, race/ethnicity, state, and urban-rural status, along with 95% confidence intervals (CIs). Age adjustment was conducted using the direct method, standardized to the year 2000 U.S. population. National trends in AAMRs were evaluated using the Joinpoint Regression Program version 5.4.0^[22]^, National Cancer Institute, which identifies statistically significant changes in trends over time using log-linear models with a maximum of five joinpoints. Annual percent change (APC) and 95% CIs were calculated. Monte Carlo permutation test was applied for significance determination. A two-tailed *P*-value of less than 0.05 was considered statistically significant.

### Ethical statement

This study was exempt from institutional review board approval as it solely relied on deidentified and publicly available data. The study adhered to the STROBE (Strengthening the Reporting of Observational Studies in Epidemiology)^[23]^ guidelines to ensure proper reporting of observational research.

## Results

A total of 284 797 deaths due to HHD with BMDs occurred between 1999 and 2020. Of these, 46.11% (*n* = 131 320) occurred at home, 25.22% (*n* = 71 837) occurred within medical facilities, 16.75% (*n* = 47 713) occurred in nursing homes/long-term care facilities, 2.77% (*n* = 7 889) occurred in hospices, and 9% occurred at other locations (*n* = 25 592). About 0.01% (*n* = 447) of deaths occurred at unknown locations.

### Annual trends for HHD with mental disorders related to AAMR

**Figure 1.**
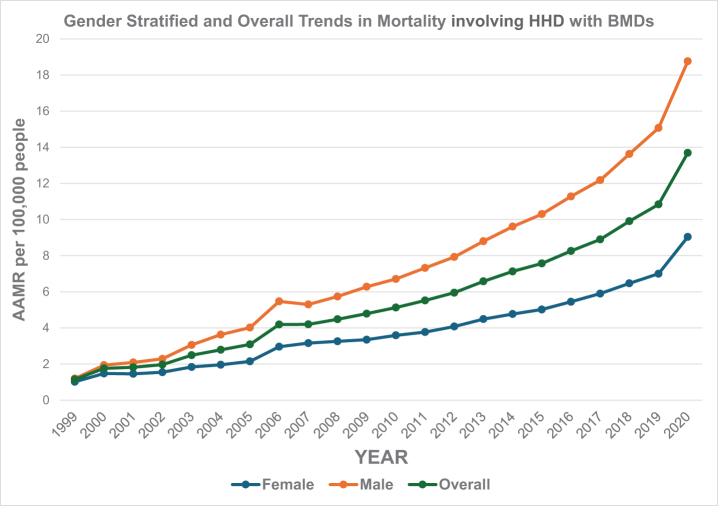
Gender stratified and overall mortality trends in the USA from 1999 to 2020 involving hypertensive heart disease and behavioral and mental disorders.

Over the study period from 1999 to 2020, the AAMR increased substantially: The AAMR in 1999 was 1.15 per 100 000, while the AAMR in 2020 increased significantly to 13.69 per 100 000. This reflects a strong upward trend over the 21-year period. The AAMR for HHD with BMDs increased steadily from 1.15 in 1999 to 4.19 in 2006 (APC: 16.29, 95% CI: 13.94–18.71; *P* < 0.000001). From 2006 to 2018, the overall AAMR for HHD with BMDs increased from 4.19 to 9.91 (APC: 7.96, 95% CI: 7.37–8.56, *P* < 0.000001). From 2018 to 2020, there was a significant increase in AAMR from 9.91 to 13.69 (95% CI; APC: 18.43; 95% CI: 12.34–24.86, *P =* 0.000008). Over the entire study period, there was an average annual percent change (AAPC) of 11.65 (95% CI: 10.73–12.58, *P* < 0.000001; Figure 1; Supplemental Digital Content Table S1, available at: http://links.lww.com/MS9/B86).

These results indicate a consistently increasing trend in mortality due to HHD with coexisting BMDs across the USA. The narrow CI and significant *P*-value confirm that the rise is both strong and reliable over time.

### AAMR stratified by sex

AAMRs were consistently higher among men compared to women throughout the study period. Among men, the AAMR increased from 1.20 in 1999 to 5.47 in 2006 (APC: 19.36%, 95% CI: 16.68–22.10, *P* **<** 0.000001). Then the AAMR further increased to 13.63 in 2018 (APC: 8.48, 95% CI: **7**.88–9.10, *P* < 0.000001). From 2018 to 2020, the AAMR has a significant increase of 13.63–18.76 (APC: 18.32, 95% CI: 12.05–24.93; *P* = 0.000011). Among women, the AAMR rose from 1.02 in 1999 to 3.16 in 2007 (APC: 13.15; 95% CI: 10.58–15.78; *P* < 0.000001). Then the AAMR further increased to 6.47 in 2018 (APC: 6.81; 95% CI: 5.80–7.83; *P* **<** 0.000001). From 2018 to 2020, the AAMR increased significantly to 9.04 (APC: 19.43; 95% CI: 9.44–30.32; *P* = 0.000652; Figure 1; Supplemental Digital Content Table S2, available at: http://links.lww.com/MS9/B86).

Over the entire study period (1999–2020), men demonstrated an AAPC of 12.93 (95% CI: 11.93–13.93; *P* **<** 0.000001), while women showed an AAPC of 10.35 (95% CI: 9.04–11.67; *P* < 0.000001). This persistent sex-based disparity in mortality indicates that men are disproportionately affected by fatal outcomes related to HHD with mental disorders. The gap widened progressively over time, with both sexes showing particularly sharp increases in the final period (2018–2020), though women showed a slightly higher APC during this last interval.

### AAMR stratified by race/ethnicity

When stratified by race/ethnicity, AAMRs were highest among Black or African American adults, followed by White, American Indian or Alaska Native, Hispanic or Latino, and Asian or Pacific Islander populations. Over the study period from 1999 to 2020, distinct patterns in AAMR were observed across all racial groups.

Among Black or African American adults, the AAMR increased markedly from 2.75 in 1999 to 6.73 in 2004 (APC: 15.75; 95% CI: 9.51–22.33; *P =* 0.000058), followed by a continued increase to 15.21 in 2018 (APC: 5.50; 95% CI: 4.72–6.28; *P* < 0.000001), and then an accelerated increase to 21.32 in 2020 (APC: 19.98; 95% CI: 9.45–31.54; *P =* 0.001027). Over the entire study period, Black or African American adults demonstrated an AAPC of 9.18 (95% CI: 7.54–10.85; *P* < 0.000001).

In White adults, the AAMR rose from 0.98 in 1999 to 3.82 in 2006 (APC: 18.28, 95% CI: 15.68–20.94; *P* < 0.000001), followed by a continued rise to 9.66 in 2018 (APC: 8.49, 95% CI: 7.81–9.18; *P* < 0.000001), and then an accelerated increase to 13.27 in 2020 (APC: 17.91, 95% CI: 11.07–25.18, *P* = 0.000038). Over the entire study period, White adults showed an AAPC of 12.55 (95% CI: 11.53–13.59; *P* < 0.000001).

For American Indian or Alaska Native adults, the AAMR increased from 1.29 in 2000 to 13.43 in 2020 (APC: 12.09; 95% CI: 10.72–13.48; *P* < 0.000001). Over the entire study period, American Indian or Alaska Native adults demonstrated an AAPC of 12.09 (95% CI: 10.72–13.48; *P* < 0.000001).

In the Hispanic or Latino population, the AAMR rose from 0.79 in 1999 to 3.23 in 2006 (APC: 17.72; 95% CI: 12.83–22.71; *P* = 0.000001), followed by a continued increase to 6.26 in 2018 (APC: 5.48; 95% CI: 4.51–6.45; *P* < 0.000001), and then a steeper increase to 10.05 in 2020 (APC: 26.30; 95% CI: 16.33–37.12; *P* = 0.000028). Over the entire study period, Hispanic or Latino adults showed an AAPC of 11.30 (95% CI: 9.61–13.02; *P* < 0.000001).

**Figure 2.**
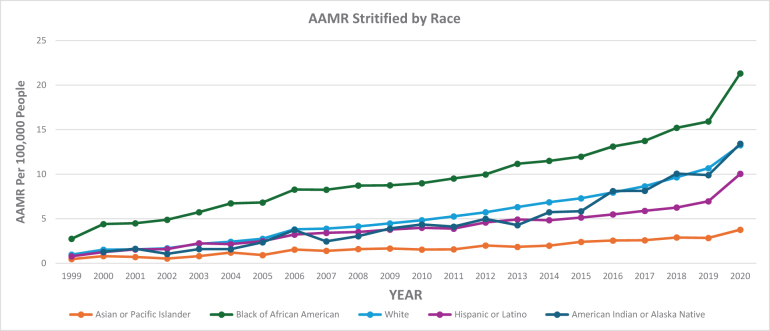
Race stratified and mortality trends in the USA from 1999 to 2020 involving hypertensive heart disease and behavioral and mental disorders.

Among Asian or Pacific Islander adults, AAMRs were the lowest throughout the study period. The rate increased from 0.48 in 1999 to 3.77 in 2020 (APC: 7.91; 95% CI: 6.88–8.94; *P* < 0.000001). Despite the low absolute values, the relative rise was significant, with an AAPC of 7.91 (6.88–8.94; *P* < 0.000001) over the entire study period (Figure 2; Supplemental Digital Content Table S3, available at: http://links.lww.com/MS9/B86).

### AAMR stratified by geographical regions

**Figure 3.**
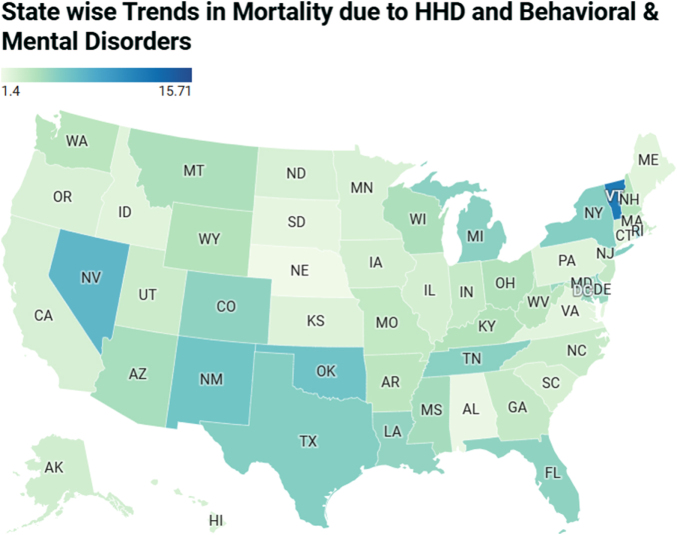
State stratified mortality trends in the USA from 1999 to 2020 involving hypertensive heart disease and behavioral and mental disorders.

The highest AAMR was found in the District of Columbia, with an AAMR of 15.71, followed by Vermont (12.07), Nevada (7.49), Rhode Island (6.62), and Oklahoma (6.52). In contrast, states with the lowest AAMRs included Nebraska (1.40), Alabama (1.61), South Dakota (1.82), Virginia (1.96), and Idaho (2.06). Among these states, the highest APC was observed in Nevada (13.09), while the lowest APC was found in the District of Columbia (4.90). This shows that the highest-burden states had AAMRs approximately 7–11 times higher than the lowest-burden states (Figure 3; Supplemental Digital Content Table S4, available at: http://links.lww.com/MS9/B86).

#### Urban areas (metropolitan regions)

In urban regions, the AAMR for HHD with BMDs rose markedly from 1.18 in 1999 to 4.48 in 2006 (APC: 17.10; 95% CI: 14.60–19.66; *P* < 0.000001), followed by a continued increase to 10.21 in 2018 (APC: 7.56; 95% CI: 6.96–8.16; *P* < 0.000001), and then an accelerated increase to 14.05 in 2020 (APC: 18.21; 95% CI: 11.54–24.83; *P* = 0.000012). Over the entire study period, urban areas demonstrated an AAPC of 11.65 (95% CI: 10.69–12.61; *P* < 0.000001). This reflects a more than 11-fold increase over the study period.

#### Rural areas (nonmetropolitan regions)

**Figure 4.**
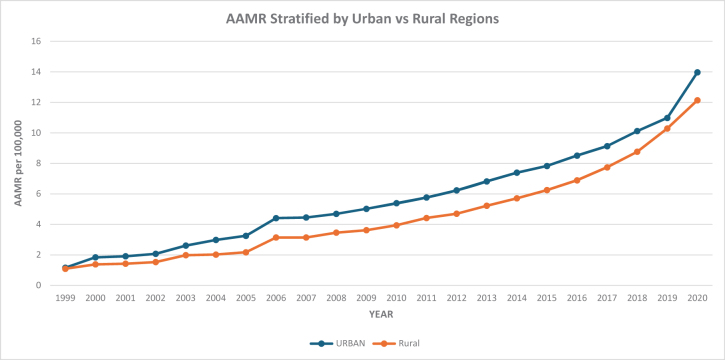
Urban/rural stratified mortality trends in the USA from 1999 to 2020 involving hypertensive heart disease and behavioral and mental disorders.

In rural areas, the AAMR rose from 1.18 in 1999 to 3.14 in 2006 (APC: 14.29; 95% CI: 12.25–16.36; *P* < 0.000001), followed by a continued increase to 7.14 in 2017 (APC: 9.36; 95% CI: 8.72–10.01; *P* < 0.000001), and then an accelerated increase to 12.17 in 2020 (APC: 17.14; 95% CI: 14.22–20.14; *P* < 0.000001). Over the entire study period, rural areas showed an AAPC of 12.08 (95% CI: 11.30–12.86; *P* < 0.000001). Although the final AAMR was slightly lower compared to urban areas, rural regions experienced a similarly steep increase in mortality (Figure 4; Supplemental Digital Content Table S5, available at: http://links.lww.com/MS9/B86).

### Census region-level variation in AAMR

A clear difference in mortality was observed among U.S. Census regions in relation to deaths caused by HHD with BMDs. The analysis used AAMR per 100 000 people and included 95% CI to reflect the precision of these estimates.

#### Census Region 3: South

This region showed the highest mortality burden. The AAMR increased from 1.45 in 1999 to 4.9 in 2006 (APC: 15.95; 95% CI: 13.31–18.67; *P* < 0.000001), followed by a continued increase to 11.19 in 2018 (APC: 7.78; 95% CI: 7.15–8.44; *P* < 0.000001), and then an accelerated increase to 16.48 in 2020 (APC: 22.18; 95% CI: 15.71–30.15; *P* = 0.000003). Over the entire study period, the Southern region demonstrated an AAPC of 11.81 (95% CI: 10.78–12.86; *P* < 0.000001). The Southern region continues to bear the greatest burden of mortality from HHD in individuals with BMDs. This elevated rate may reflect persistent challenges such as reduced access to healthcare, higher prevalence of chronic disease, and limited mental health support systems.

#### Census Region 1: Northeast

The second-highest mortality rates were observed here. The AAMR increased from 0.99 in 1999 to 4.39 in 2006 (APC: 18.86; 95% CI: 14.36–23.53; *P* < 0.000001), followed by a continued increase to 12.58 in 2020 (APC: 7.63; 95% CI: 6.93–8.36; *P* < 0.000001). Over the entire study period, the Northeast region showed an AAPC of 11.25 (95% CI: 9.85–12.66; *P* < 0.000001). Although the Northeast has more healthcare infrastructure, the AAMR remains elevated, possibly due to an older population and better detection of coexisting mental health conditions alongside CVD.

#### Census Region 2: Midwest

The Midwest had moderate mortality rates. The AAMR increased from 1.06 in 1999 to 3.64 in 2007 (APC: 15.05; 95% CI: 12.35–17.81; *P* < 0.000001), followed by a continued increase to 9.0 in 2018 (APC: 8.84; 95% CI: 7.81–9.88; *P* < 0.000001), and then an accelerated increase to 12.51 in 2020 (APC: 16.50; 95% CI: 7.25–26.54; *P* = 0.001415). Over the entire study period, the Midwest region demonstrated an AAPC of 11.89 (95% CI: 10.57–13.22; *P* < 0.000001). This region sits between the higher rates of the South and Northeast and the lower rates seen in the West. While access to care may be somewhat better than in the South, rural areas in the Midwest still face challenges in managing chronic and mental health conditions.

#### Census Region 4: West

**Figure 5.**
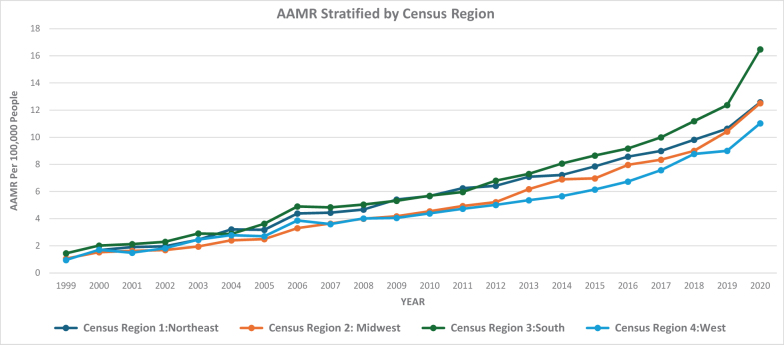
Census region stratified mortality trends in the USA from 1999 to 2020 involving hypertensive heart disease and behavioral and mental disorders.

This region had the lowest mortality rates. The AAMR increased from 0.96 in 1999 to 3.87 in 2006 (APC: 16.61; 95% CI: 11.76–21.68; *P* = 0.000002), followed by a continued increase to 6.15 in 2015 (APC: 6.07; 95% CI: 4.04–8.12; *P* = 0.000012), and then an accelerated increase to 11.03 in 2020 (APC: 12.33; 95% CI: 9.29–15.463; *P* < 0.000001). Over the entire study period, the Western region showed an AAPC of 10.98 (95% CI: 9.20–12.78; *P* < 0.000001). The lower rate in the West may reflect regional differences in health behavior, better outpatient care, and preventive health efforts. However, disparities likely remain in specific underserved areas (Figure 5; Supplemental Digital Content Table S6, available at: http://links.lww.com/MS9/B86).

**Figure 6.**
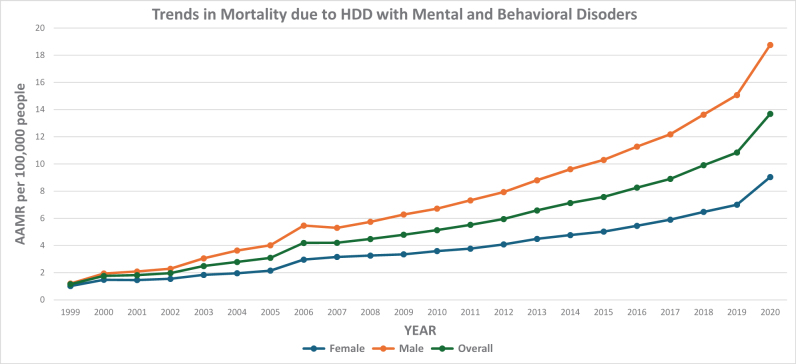
Central illustration.

Overall, these findings highlight regional disparities in health outcomes related to both CV and mental health, suggesting a need for targeted public health strategies in the most affected areas (Figure 6)

## Discussion

Our study presents a comprehensive analysis of mortality trends from 1999 to 2020 from the Centers for Disease Control and Prevention among individuals with HHD and BMDs. It gave several important findings.

In this study of 284 797 deaths attributable to HHD and BMDs, the overall AAMR showed a significant increase from 1999 to 2020. First, there was an initial period of increase in mortality rates from 1999 to 2006, following which a progressive increase was noted until 2020. Throughout the period, males consistently exhibit higher mortality rates than females, despite both starting from almost the same initial values (1.02 in 1999 to 9.04 in 2020 in women as compared to 1.20 in 1999 to 18.76 in 2020 in men).

Considering racial groups, Black or African American adults were at the highest risk of death from HHD and coexisting BMDs, followed by White, American Indian or Alaska Native, Hispanic or Latino, and Asian or Pacific Islander populations. Significant regional differences were observed, with states in the top 90th percentile (District of Columbia, Vermont, Nevada, Rhode Island, and Oklahoma) having approximately 5.5 times higher AAMRs compared with states in the lower 10th percentile (Alabama, South Dakota, Virginia, Nebraska, and Idaho), highlighting a significant disparity across states. Overall, the Southern region had the highest AAMR, followed by the Northeast, Midwest, and West. The Southern region continues to bear the greatest burden of mortality from HHD in individuals with BMDs. This elevated rate may reflect persistent challenges such as reduced access to healthcare, higher prevalence of chronic disease, and limited mental health support systems. The lower rate in the West may reflect regional differences in health behavior, better outpatient care, and preventive health efforts. While access to care may be somewhat better in regions other than the South, underserved areas in the Northeast, Midwest, and West still face challenges in managing chronic and mental health conditions.

Moreover, urban areas (metropolitan regions) demonstrated slightly higher mortality rates compared with rural areas (nonmetropolitan regions). Although the final AAMR was slightly lower in rural areas compared to urban areas, rural regions experienced a similarly steep increase in mortality. This trend may reflect ongoing issues with healthcare access, underdiagnosis or delayed diagnosis of mental disorders, limited CV care services, and socioeconomic barriers in rural communities. The findings suggest a significant health burden in rural areas, albeit slightly lower than in urban counterparts by the end of the study period. Overall, these findings highlight regional disparities in health outcomes related to both CV and mental health, suggesting a need for targeted public health strategies in the most affected areas.

Mental disorder amplifies the mortality risk in hypertension significantly, showing that individuals with both hypertension and common mental disorders (e.g., depression/anxiety) had significantly higher CV mortality (hazard ratio 2.32 for controlled hypertension) compared with normotensive individuals without mental disorders^[24]^. Hypertension and depression alone are related to mortality independently, but the co-existence of both increases the risk of mortality^[25]^. It had been previously reported even before 1999 that hypertension with depression had higher mortality as compared to non-depressed hypertensive patients^[26]^. In order to enhance outcomes, it is indicated that individuals with depression and hypertension should be rigorously monitored^[25]^. Several explanations have been proposed for the excess mortality in these groups, including the exacerbation of CV risks due to mental health conditions in hypertensive individuals^[24]^, the prevalence of substance abuse^[20]^, side effects of pharmacological treatments^[27]^, unhealthy lifestyles, smoking, low socioeconomic status, and demographic disparities.

Patients with psychiatric disorders are 3-fold more likely to smoke^[13]^. Side effects of pharmacological treatments, e.g., antidepressants, would lead to the presence of hypertension in depressive patients. Tricyclic antidepressants and monoamine oxidase inhibitors have been linked to alterations in both systolic and diastolic blood pressure^[28]^. A meta-analysis revealed an increase in blood pressure that depends on the dosage of venlafaxine administered^[29]^. Individuals diagnosed with major depressive disorder tend to have considerably poorer lifestyle habits and exhibit more physiological disruptions than healthy individuals, which in turn elevates their chances of developing CV issues and increases mortality risk by approximately 80%^[8]^. There is a two-way spiral relationship between hypertension and mental disorders leading to increased prevalence of hypertension in psychiatric patients and vice versa and thus causing increased mortality^[30,31]^. Adults in the USA experiencing symptoms of anxiety or depression and living on a low income are more likely to suffer from hypertension compared to those without such symptoms and are associated with comorbidities. Individuals who reported using medication for anxiety disorders or depression had a greater likelihood of being diagnosed with hypertension. When treating patients with hypertension or anxiety and depression, particularly those with lower socioeconomic status, it is increasingly essential to explore the connections among mental health, hypertension, and socioeconomic status^[32]^. Moreover, patients with BMDs generally have reduced access to healthcare due to socioeconomic conditions^[20]^ along with adverse perceptions from healthcare professionals in non-mental health specialties toward individuals with serious mental illnesses and the detrimental social effects associated with possessing a mental disorder^[33,34]^. Studies show that patients with depression are less likely to effectively manage their hypertension^[35]^, yet greater healthcare utilization among these patients may contribute to faster hypertension control and less CVD mortality^[36]^. But this causes a higher economic burden of healthcare utilization than those without mental health disorders^[37]^. The prevalence of hypertension is also higher in specific psychiatric populations, such as those with bipolar disorder and schizophrenia, which may contribute to worse clinical outcomes^[38]^. In the context of gender variations, men with mental disorders and hypertension show a greater increase in CV mortality compared to a lesser increase in women. This disparity may be attributed to men’s reduced healthcare utilization^[39]^, more substance and opium abuse in males^[40]^, poorer medication adherence^[41]^, poorer dietary habits^[42]^, higher alcohol consumption^[43]^, and a higher rate of smoking in men^[44]^. Primary prevention methods, including the embrace of various healthy lifestyle habits and the utilization of established medications, are typically more common among women than men^[45,46]^.

Overall, although there was a notable rise in mortality among White patients, we noted consistently higher mortality rates throughout the study period among Black patients. This observation can mainly be attributed to variations in socioeconomic status among different racial groups, risk factors for CVD, a greater prevalence of comorbidities, and limited access to healthcare^[47]^. In a recent study, similar findings were observed in the Black population, highlighting several social factors such as unemployment, low family income, food insecurity, lack of home ownership, and being unpartnered. Additionally, behavioral factors like current smoking, insufficient leisure-time physical activity, and sleep durations of less than 6 or more than 8 hours per day, along with metabolic risk factors such as obesity, hypertension, and diabetes, were associated with a significantly increased risk of CVD mortality^[48,49]^. The identified racial/ethnic differences in mortality may occur due to structural and socioeconomic reasons or factors. Limited access to treatment for hypertension, unequal access to evaluation and treatment for mental health issues, and poor adherence to medication may all contribute to these patterns of mortality. Public health interventions that include equitable access to preventive care, culturally adapted mental health interventions, and screening for illness in the early stages may help to reduce disparities over time.

We observed a significantly prominent increase in mortality in patients with hypertension and coexisting BMDs in both urban and rural areas, with slightly increased mortality in urban areas, as exposure to environmental stressors, such as air pollution and noise, is higher in urban areas and has been linked to increased risk of CV events and can exacerbate anxiety and depression. The prevalence of substance abuse-related mental disorders, which significantly increase CV mortality, is also higher in urban populations^[20]^. Meanwhile, in rural areas, there has been a disproportionate number of hospital closures, making it challenging for patients with HHD and coexisting BMDs to receive management for CVD and mental disorders in these communities, leading to higher mortality in these areas^[50]^.

Additional prospective longitudinal research involving a large cohort of patients with hypertension and coexisting BMDs may be necessary to confirm our findings and to investigate the factors that will reduce all-cause and CV mortality in this population.

## Study limitations

This study, like other epidemiological analyses, has a number of limitations that should be acknowledged. First, since the study relies on ICD codes and death certificates, there is a chance that HHD or BMDs were incorrectly recorded or overlooked as causes of death. Second, the database does not provide clinical details like vital signs, lab tests, heart function measurements, or genetic data that are important for fully describing HHD. Similarly, another limitation is the absence of detailed clinical information related to BMDs, including diagnostic criteria, treatment history, medication adherence, and co-occurring psychiatric or neurological conditions. Third, a further limitation is that treatment information, including drugs or interventions for managing hypertensive heart disease or mental health conditions, is not available in the dataset. Fourth, our findings should be interpreted as representing deaths involving HHD with coexisting BMDs, rather than deaths exclusively due to HHD. Fifth, we analyzed deaths involving any coexisting BMDs (F01–F99) as a single category; stratification by specific disorder subgroups was not performed due to small counts in several strata, which could lead to unstable estimates. Sixth, the observed steepest increase in 2020 coincides with the COVID-19 pandemic, during which coding practices and access to care may have been disrupted, potentially influencing mortality trends. Finally, there was no information on socioeconomic determinants of health, which could have an impact on healthcare access.

## Conclusion

Between 1999 and 2020, the number of deaths in the USA caused by HHD combined with BMDs increased dramatically, with AAMR rising by more than 10-fold, from 1.15 to 13.69 deaths per 100 000 people. Men were always found to have larger mortality rates than women, and the burden was highest among the Black or African American adults in all the races. They also found that mortality rates were highest in the Southern census region, as well as within states, including the District of Columbia and Vermont, indicating regional inequalities that may be linked to variations in healthcare accessibility, socioeconomic privileges, and comorbidity load. By 2020, the mortality rate had increased in urban regions a bit more than in rural ones, although the increase was alarming in both of them. In the future, attention should be drawn to root causes, with integrated yet targeted policy-level interventions and robust screening recommendations prioritized to address this increasing mortality burden as an urgent public health imperative.

## Data Availability

None.

## Research registration unique identifying number (UIN)

N/A.

## Transparency statement

This document was prepared with a commitment to transparency, accuracy, and integrity in research. All data, methodologies, and analyses included in this research have been extracted from CDC WONDER database, the official website of the government of the USA. All the data have been meticulously extracted and reviewed. Any potential conflicts of interest have been disclosed and all efforts have been made to ensure that the findings presented are unbiased and based on robust evidence. The authors have adhered to the ethical guidelines for research and publication, including maintaining objectivity and providing a clear and honest account of the research conducted. All sources of funding, if applicable, have been disclosed, and there has been no undue influence from sponsors or external parties in the preparation of this document. We encourage peer review and welcome constructive feedback to further enhance and refine the reliability of this work.
